# Virtual Family-Centered Rounds in the Neonatal Intensive Care Unit: Protocol for a Cluster Randomized Controlled Trial

**DOI:** 10.21203/rs.3.rs-2644794/v1

**Published:** 2023-04-17

**Authors:** Jennifer L. Rosenthal, Daniel J. Tancredi, James P. Marcin, Audriana Ketchersid, Elva T. Horath, Erika N. Zerda, Trevor R. Bushong, Daniel S. Merriott, Patrick S. Romano, Heather M. Young, Kristin R. Hoffman

**Affiliations:** a Department of Pediatrics, University of California Davis, 2516 Stockton Blvd, Sacramento, CA 95817; b Center for Health and Technology, University of California Davis, 4610 X Street, Sacramento, CA 95817; c Department of Internal Medicine and Center for Healthcare Policy and Research, University of California Davis, 4150 V St, Sacramento, CA 95817; d Betty Irene Moore School of Nursing, University of California Davis, 2570 48th St, Sacramento, CA 95817

**Keywords:** Pediatrics, Neonatal Intensive Care Units, Neonate, Clinical Trial, Telemedicine, Patient-Centered Care, Patient Reported Outcome Measures

## Abstract

**Background:**

Family-centered rounds is recognized as a best practice for hospitalized children, but it has only been possible for children whose families can physically be at the bedside during hospital rounds. The use of telehealth to bring a family member virtually to the child’s bedside during rounds is a promising solution. We aim to evaluate the impact of virtual family-centered rounds in the neonatal intensive care unit on parental and neonatal outcomes.

**Methods:**

This two-arm cluster randomized controlled trial will randomize families of hospitalized infants to have the option to use telehealth for virtual rounds (intervention) or usual care (control). The intervention-arm families will also have the option to participate in rounds in-person or to not participate in rounds. All eligible infants who are admitted to this single-site neonatal intensive care unit during the study period will be included. Eligibility requires that there be an English-proficient adult parent or guardian. We will measure participant-level outcome data to test the impact on family-centered rounds attendance, parent experience, family-centered care, parent activation, parent health-related quality of life, length of stay, breastmilk feeding, and neonatal growth. Additionally, we will conduct a mixed methods implementation evaluation using the RE-AIM (Reach, Effectiveness, Adoption, Implementation, Maintenance) framework.

**Discussion:**

The findings from this trial will increase our understanding about virtual family-centered rounds in the neonatal intensive care unit. The mixed methods implementation evaluation will enhance our understanding about the contextual factors that influence the implementation and rigorous evaluation of our intervention.

**Trial registration:**

ClinicalTrials.gov Identifier: NCT05762835. Status: Not yet recruiting. First Posted: 3/10/2023; Last Update Posted: 3/10/2023.

## Introduction

### Background and rationale {6a}

Family-centered rounds (FCR) are bedside rounds for hospitalized patients that engage families as active members of the multidisciplinary team.^1^ Benefits of FCR include fewer harmful errors, shortened hospital stays, reduced parental anxiety, better family understanding, improved family experience, and enhanced staff teamwork.^2–8^ FCR is the most commonly reported rounding model in pediatric hospital settings^8^ and is recognized as a best practice for hospitalized children.^9,10^ Despite its widespread adoption, FCR has only been possible for hospitalized children whose families can physically be at the bedside during rounds.

The barriers that limit family presence at the bedside are particularly challenging for families with critically ill infants hospitalized in the neonatal intensive care unit (NICU). These infants often experience prolonged hospitalizations in regional referral centers located far from their parents’ or guardians’ (“parents” hereafter) residence.^11^ Travel, financial, work, or childcare challenges limit parents’ ability to be physically present in the NICU.^12,13^ These challenges particularly impact rural and low-income families.^14^

The value of parent FCR attendance is especially important in the NICU. Parents of critically ill infants have high rates of depression, anxiety, and post-traumatic stress.^15–18^ Separation of the parent-infant dyad can worsen parental depression and hinder parental-newborn attachment,^16^ which can have harmful consequences on the child’s future intellectual development and wellbeing.^19^ Strategies are therefore needed to support parents’ ability to attend FCR while their infant is hospitalized in the NICU.

The use of telehealth to bring a parent virtually to the child’s bedside in the NICU to participate in FCR has the potential to promote more family-centered care. In this protocol report, telehealth refers to the use of live, bidirectional communications between patient families and healthcare professionals using HIPAA-compliant audiovisual telecommunication technologies.^20^ Telehealth use may increase family-centeredness of care by mitigating the challenges parents encounter that prevent them from attending FCR. Prior pediatric and adult research suggests that virtual FCR is meaningful to patients, families, and clinicians.^21–24^ Prior research has examined virtual FCR in non-NICU settings using non-randomized designs.^21,22,25–29^ Several studies have brought NICU providers virtually to the bedside,^30–34^ but a pilot trial conducted by our research team^24^ was the first clinical trial to bring family members virtually to the NICU bedside. Our pilot trial supported the feasibility of conducting a randomized trial to compare virtual FCR to usual care in the NICU; however, it was limited in that it lacked power for hypothesis testing, had limited outcomes measured, and was conducted in 2020 during COVID-19 shelter-in-place orders. Therefore, we now propose a rigorous virtual FCR trial to understand the impact of telehealth on parental and infant outcomes.

### Objectives {7}

Our central hypothesis is that virtual FCR may improve infant and parent outcomes by optimizing FCR. Specifically, we hypothesize that virtual FCR may increase FCR parent attendance, improve parent experience and family-centeredness of care, increase parent activation, improve parent health-related quality of life (HRQOL), shorten NICU length of stay, and improve breastmilk feeding and neonatal growth. We therefore aim to evaluate the impact of virtual FCR on these parental and infant outcomes.

### Trial design {8}

**Figure 1:**
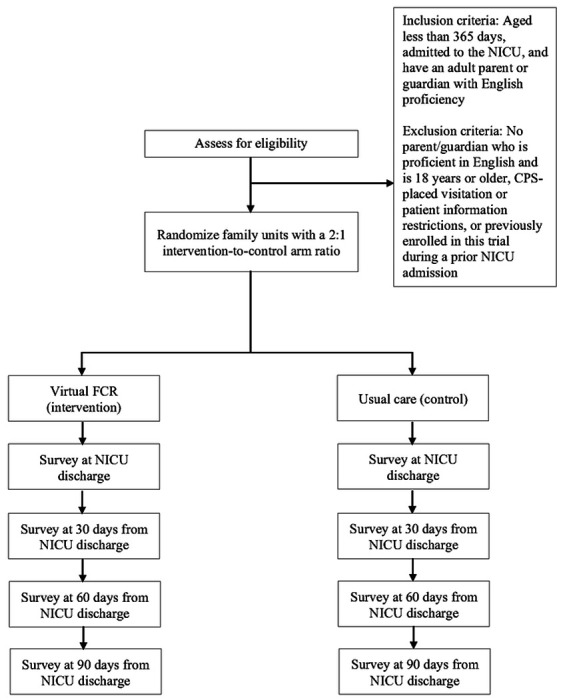
Overview of the trial procedures FCR – family-centered rounds NICU – neonatal intensive care unit CPS – child protective services

This study will use a two-arm cluster randomized controlled trial design. The cluster is the family unit, which is any combination of infants resulting from the same pregnancy and receiving care in the same NICU. Figure 1 shows the overview of the trial procedures. We will examine parental and infant outcomes. Additionally, we will conduct a mixed methods implementation evaluation using the RE-AIM (Reach, Effectiveness, Adoption, Implementation, Maintenance) framework^35^ to understand how to optimize the translation of our intervention across diverse groups and settings and facilitate the translation of our research into practice.^36^ This trial protocol follows the SPIRIT (Standard Protocol Items: Recommendations for Interventional Trials) guideline.^37^

## Methods

### Study setting {9}

The hospital is a 121-bed metropolitan quaternary care children’s hospital within a university hospital, which serves as a referral center for infants across a 33-county region spanning 65,000 square miles. The level IV NICU has 49 beds, admits over 900 neonatal patients annually, and routinely receives neonatal transfers from 30 hospitals in the region.

### Eligibility criteria {10}

Eligible patients will be infants aged less than 365 days who are admitted to the NICU and have an adult parent or guardian with English proficiency. This trial will exclude infants if they have restrictions placed by child protective services, including visitation restrictions or restricted access to patient information. Infants with more than one NICU admission during the trial period will only be included on their first admission. We limit this trial to parents with English proficiency because we will use an existing telehealth platform that is only available in English. We will first test the efficacy of this intervention before adapting the platform’s family-facing interface into additional languages.

All eligible infants will be enrolled in the study. Enrollment will occur during weekdays by a research assistant. Exceptions to enrollment of eligible infants will be for those infants with very brief NICU stays that are admitted and discharged on a weekend (or during other periods of time when a research assistant is not working).

### Who will take informed consent? {26a}

This research has a waiver of consent for the intervention because telehealth use for FCR is an existing clinical resource that can be used for all hospitalized patients. No NICU care team member or parent will be required to use the intervention. The parent survey that will be used for data collection of parent-reported outcomes will have elements of informed consent before the survey questions. Following the informed consent information, there will be a statement that if the participants agrees to take part in the research, to please proceed by answering the following questions.

### Additional consent provisions for collection and use of participant data and biological specimens {26b}

No biological specimens will be collected for storage.

#### INTERVENTIONS

##### Explanation for the choice of comparators {6b}

The two arms of the trials are: (1) Intervention arm – virtual FCR plus usual care; (2) Control arm – usual care. Subjects assigned to the control arm will receive usual care. We seek to identify if our intervention is superior to usual care, because we recognize that although FCR is considered best practice for hospitalized children, the practice of FCR across different providers and settings may be highly variable. Control-arm parents will have the option to attend FCR in person or to not attend FCR.

##### Intervention description {11a}

The NICU team members will use a computer with a speaker and pan-tilt-zoom camera, mounted on a stand with wheels to launch telehealth connections using the secure application called ExtendedCare. The ExtendedCare platform meets Health Insurance Portability and Accountability Act security rules and launches from the patient’s EHR. From within this telehealth connection, a NICU team member will send an electronic message (e.g. via text or email) to the parent(s) and wait for the parent to join the visit to establish a secure videoconference. The message to the parent includes a link that can be clicked to open a browser that allows the parent to join the telehealth visit. The parent does not need to download or use any application or program. FCR will then proceed in usual fashion with the NICU team members and – if in attendance – parent(s). Parents and NICU team members will have a 24/7 helpdesk number to call to report and troubleshoot any technical issues. The NICU team members attending FCR typically include a neonatologist, neonatal fellow, neonatal nurse practitioners, pediatric residents, charge nurse, bedside nurse, respiratory therapist, pharmacist, dietician, and social worker.

##### Criteria for discontinuing or modifying allocated interventions {11b}

Parents of intervention arm subjects can choose to engage or not in the virtual FCR intervention.

They can participate in virtual FCR as much, or as little, as they choose. Parents also will have the option to attend FCR in person or to not attend FCR.

In the event that parents of control arm subjects ask to use telehealth to attend FCR virtually, we will provide them access. We will record these protocol assignment deviations and categorize these subjects as a “crossover.”

##### Strategies to improve adherence to interventions {11c}

Parents of family units assigned to the intervention arm will be invited to sign up for virtual FCR. Parents accepting this offer will be considered “subscribed” and will provide their preferred method of contact information (cell phone number or email address) to receive a secure link to join FCR virtually every weekday morning. Efforts to subscribe parents will consist of in-person, secure text message, or phone call outreach. For subjects with more than one parent listed in the EHR, separate invitations will be sent to each parent. Three attempts will be made per parent within the first three days of trial enrollment, or until the parent declines or accepts the invitation. Invitations will also be sent every 14 days; we learned during our pilot trial that parents who initially decline sometimes change their decision and appreciate outreach that extends throughout the NICU hospitalization.

We will apply standard steps of quality improvement^38^ to conduct a series of Plan-Do-Study Act (PDSA) cycles targeting intervention subscription, intervention adherence, and survey response measures and use statistical process control methods^39^ to evaluate our tested strategies. We will plot the measures in time series fashion on separate Shewhart charts. We will determine when a strategy is associated with meaningful change using these steps: (1) calculate sigma to establish upper/lower control limits, (2) plot weekly [subscription and adherence] data or monthly [survey response] data sequentially, (3) meet weekly to evaluate the charts for special cause variation.^39^

##### Relevant concomitant care permitted or prohibited during the trial {11d}

Other interventions related to FCR that are delivered during the NICU hospitalization will be prohibited during the trial. Usual care practices will be permitted during the trial, including practices already in place that enhance family-centered care such as the NICU video viewing program. The video viewing program is available for all families in the NICU to watch real-time video of their infants. This program is not bidirectional, and it does not include audio.

##### Provisions for post-trial care {30}

Post-trial care is not required for this minimal risk trial.

##### Outcomes {12}

Table 1 shows an overview of the trial outcome measures.

###### Primary:

The primary outcome is FCR parent attendance, which will be defined at the family unit level, accounting for the possibility of multiple enrolled infants per family and variable lengths of stay for each infant. FCR parent attendance is the primary outcome, because successfully increasing parent FCR attendance would increase the delivery of best practice care. We will compute the total number of possible weekday FCR encounters per family (the ‘denominator’) and the number of those for which at least one parent was present virtually or in-person (the ‘numerator’). For example, if a family has two infants, one whose length of stay includes five FCR encounters and another whose length of stay includes seven FCR encounters, that family will be counted as having twelve possible FCR encounters. If at least one parent is present for four of the first infant’s encounters and six of the second infant’s encounters, the family would be counted as having attended ten of the twelve FCR encounters. In practice, for families with two or more hospitalized infants, the NICU team typically conducts rounds for each infant together (or in sequence); however, this practice is not guaranteed and there might be occasions when a parent is unable to attend FCR for all their infants. For other analyses (e.g., when FCR parent attendance is being modeled as a mediator or modifier or other outcomes), we will define alternative numerators and denominators that are appropriately specific to either an infant and/or a parent, using straightforward alterations of the above rules.

###### Secondary:

Parent experience will be assessed using the two items measuring overall experience from the Child Hospital Consumer Assessment of Healthcare Providers and Systems (HCAHPS) Survey.^40–41^ Family-centered care will be assessed using the Family-Centered Care Experience (FACCE) survey.^42^ Parent activation will be assessed using the Parent-Patient Activation Measure (P-PAM).^43,44^ Parent HRQOL will be assessed using the PedsQL^™^ Family Impact Module.^45^ The Family Impact Module consists of the following subscales: physical functioning, emotional functioning, social functioning, cognitive functioning, communication, worry, daily activities, and family relationships.^45^ Every eligible parent will receive these parent-reported instruments at the time their infant is discharged from the NICU. Parents will receive the instrument measuring HRQOL at 0, 30, 60, and 90 days from that discharge date. For a family unit with multiple enrolled infants, the distribution timing of the parent-reported instruments will be based on the last discharge date among their infants.

NICU length of stay (days) will be obtained from the electronic health record (EHR). Measures of breastmilk feeding will be dichotomous outcomes and include breastmilk feeding initiation, any breastmilk feeding at the time of discharge from the NICU (and 90 days later), and exclusive breastmilk feeding at the time of discharge from the NICU (and 90 days later). Breastmilk feeding includes consuming milk from the birth parent via any delivery method (e.g., bottle, feeding tube, breast). Any breastmilk feeding will be defined as the infant consuming any amount of milk from the birth parent, with or without the addition of formula or fortifier. Exclusive breastmilk feeding will be defined as 100% of base feeding type as milk from the birth parent, with or without a bovine or human fortifier. Breastmilk feeding at discharge and 90 days later will be obtained from the EHR and parent survey, respectively.

Postnatal growth failure will be assessed at NICU discharge using sex-specific Fenton growth charts and expressed as both a dichotomous variable and a categorical variable. The dichotomous outcome will define growth failure as a weight-for-gestational-age Z-score decline of more than 0.8 standard deviations (SD) from birth to discharge.^46,47^ For the categorical outcome, the degree of growth failure will be classified as none (no decline or a decline £0.8 SD), mild (>0.8 and £1.2 SD), moderate (>1.2 and £2 SD), or severe (>2 SD).

###### Exploratory:

Neonatal growth velocity will be measured as an exploratory outcome. We will use the sex-specific Fenton growth charts to calculate change in Z-score divided by number of days in the NICU.^48^

Adverse events and errors will be collected using an established process^49–52^ that involves review of data from EHR and solicited reports. Two neonatologists, blinded to the study arm, will independently categorize each event as a harmful error (preventable adverse event), non-harmful error, non-preventable adverse event, or exclusion.^5^ Our team successfully used this procedure in our pilot trial testing virtual FCR in the NICU (74.3% agreement; kappa, 0.59; 95% CI 0.53–0.66).^24^ Outcomes measures will include the rates of harmful errors, non-harmful errors, and overall errors (harmful errors plus non-harmful errors). We will additionally include 30-day post-discharge revisits to any emergency department and unplanned readmissions to any hospital, which will be obtained by chart review and parent-reported survey.

Exploratory outcomes will also include existing NICU metrics collected for the California Perinatal Quality Care Collaborative,^53^ including temperature instability, central line-associated bloodstream infection, central line days, and antibiotics days. Temperature instability will be a dichotomous variable defined as any occurrence of a temperature below 36°C during the NICU hospitalization. Central line-associated bloodstream infection will be a dichotomous variable defined as any occurrence during the NICU hospitalization of a laboratory-confirmed bacterial or viral bloodstream infection that develops with a central line in place and is not related to an infection at another site. Central line days will be the number of days among the total number of NICU days that the infant has an umbilical catheter or one or more central lines in place. Antibiotics days will be the number of days among the total number of NICU days that the infant receives intramuscular or intravascular antibacterial or antifungal agents.

##### Participant timeline {13}

Table 2 shows the schedule of enrollment, intervention, and assessments.

##### Sample size {14}

We specified effect sizes of interest for all the primary and secondary outcomes trial outcomes and determined the sample size needed to satisfy each. The sample size estimation that satisfies length of stay requirements satisfies requirements for all other primary and secondary outcomes of interest. Outcome distributional assumptions were based on our pilot trial^24^ data or other literature. Pilot trial baseline mean log transformed length of stay ± standard deviation (SD) was 3.0 ± 1.0 days. Minimally clinically important differences (MCID) were based on literature when available or via consensus opinion elicited from NICU clinicians. Experts specified that a 25% reduction in geometric mean length of stay was of interest. A sample of 447 family units subject to 4% attrition in each arm yields a sufficient sample (429 family units) to provide 80% power (2-sided testing, alpha=5%) to detect this MCID. These power calculations conservatively assumed one infant per family unit and one parent survey respondent per family unit; however, we will likely enroll more than one infant and more than one parent per family unit. The attrition estimate is based on 4 of 110 randomized infants in the pilot trial being excluded from final analysis (e.g., child protective visitation restrictions placed after randomization). Parent-reported outcomes assume a 75% survey response rate.

##### Recruitment {15}

A research assistant will identify all eligible subjects using the EHR – with clarification from the NICU care team, as needed – on every newly admitted infant to the NICU. Adequate participant enrollment to reach the target sample size is feasible given the procedure that all eligible infants will be enrolled in the study.

#### ASSIGNMENT OF INTERVENTIONS: ALLOCATION

##### Sequence generation {16a}

The unit of randomization will be the family unit. We will randomize at the family level rather than the infant patient level so that parents of twins or other multiples have their children assigned to the same study arm. A study statistician will generate a random allocation list in Stata and employ allocation concealment to assign eligible subjects with a 2:1 intervention-to-control arm ratio.

##### Concealment mechanism {16b}

A person not involved in recruitment will upload the randomization allocation list to the randomization module in REDCap; this module ensures that the sequence is concealed until the study arm is assigned. We will use unequal randomization rather than equal allocation, because although it requires a 12.5% increase in the total sample size to maintain precision of between arm effect size estimates, it yields a 50% larger number of subjects in the intervention arm. Having more individuals in the intervention-arm will permit more opportunities to examine intervention implementation outcomes.

##### Implementation {16c}

A research assistant will electronically open the sequentially numbered assignments using the REDCap randomization module. The research assistant will invite parents of family units assigned to the intervention arm to sign up (“subscribe”) to use virtual FCR. They will also solicit assistance from the NICU care team to subscribe parents.

#### ASSIGNMENT OF INTERVENTIONS: BLINDING

##### Who will be blinded {17a}

Trial participants and NICU care team members will not be blinded. The two neonatologists who will independently categorize potential adverse events and errors will be blinded to the study arm.

##### Procedure for unblinding if needed {17b}

Not applicable. The infants/parents and NICU care team members will not be blinded.

#### DATA COLLECTION AND MANAGEMENT

##### Plans for assessment and collection of outcomes {18a}

Data collection for the outcomes will include EHR chart review and parent surveys. For infants with more than one parent listed in the EHR, separate surveys will be distributed to each parent. Parent respondents will include only English-proficient adults. We will not send survey packets to parents of infants who die during the NICU hospitalization.

EHR data will be used to abstract patient characteristics (estimated gestational age at birth, inborn versus outborn delivery, day of life on admission, race, ethnicity, sex, insurance, invasive ventilator days, diagnoses, residence-to-NICU distance, California Healthy Places Index^54^, and NICU disposition). Demographic characteristics of parents (age, race, ethnicity, gender, relationship to the infant, education, employment, transportation security, marital status, housing, other children dependents, computer/smart device access, internet access, and digital literacy score^55^) will be collected in the survey packets.

##### Plans to promote participant retention and complete follow-up {18b}

Participation in using virtual FCR is voluntary and refusal to participate will involve no penalty to patients, parents, or NICU care team members. Regarding parent surveys, participant outreach will include in-person, text/email, or phone recruitment. The survey packet will be sent four times; surveys not completed within 21 days of distribution will be considered non-response. Participants will receive a $15 gift card for each survey packet that they return. Parents completing survey packets at 0, 30, 60, and 90 days will therefore receive a total of $60 in gift cards.

##### Data management {19}

Surveys and EHR data will be stored in REDCap on a secure server. To promote data quality, REDCap data entry fields will include required fields, range checks for data values, and instructions for data entry procedures.

##### Confidentiality {27}

This study was deemed by the University of California Davis Institutional Review Board to only involve minimal risk related to the potential loss of confidentiality. Only authorized research team persons will be granted access to personal information about potential and enrolled participants. Identifiers stored on computers will be encrypted and password protected. All data will be destroyed seven years after completion of the study.

##### Plans for collection, laboratory evaluation and storage of biological specimens for genetic or molecular analysis in this trial/future use {33}

This trial does not require **collection, laboratory evaluation and storage of biological specimens**.

#### STATISTICAL METHODS

##### Statistical methods for primary and secondary outcomes {20a}

Our primary analysis strategy will analyze all available data from participating family units and their members, with groups defined according to the randomized assignment (intervention versus control) for that family unit. We will use graphical and analytical descriptive statistics to summarize infant, parent, and family unit characteristics. We will use methods for clustered survey data^56^ to adjust confidence intervals for family units with multiple parental respondents. We will not use cluster-adjusted confidence intervals for family units with multiple infants unless the mean number of infants per family unit unexpectedly exceeds 1.10.

We will use generalized linear models to estimate intervention effect sizes and confidence intervals and to test hypotheses for outcomes. Independent variables will include a binary indicator for intervention assignment along with a parsimonious set of subject characteristics as covariates (parent age, race, ethnicity, gender, education, transportation security, marital status, other children dependents, digital literacy score^55^). We will specify the generalized linear mixed models as linear, logistic, or Poisson regressions, according to the outcome type. Random effects will be specified to accommodate the multilevel structure of the data and the nesting of longitudinal measurements, when applicable, within parents and the nesting of parents and infants within family units.

To account for increased exposure among subjects with longer NICU stays when analyzing the primary outcome, we will use Poisson regression to compare rates for the FCR parent attendance outcome between intervention vs. control group subjects, using the numerator for this outcome as the dependent variable and using the logarithm of the denominator as an offset term. We will also use Poisson regression for analyzing the error rate outcomes, with the offset being the logarithm of the number of days in the NICU. The exponentiation of the Poisson regression coefficient for the treatment indicator will thus represent an adjusted between-arm rate ratio for FCR parent attendance.

For parent experience and family-centered care, we will use the top-box scoring method^40,41^ and assess intervention effects on the individual items as well as on the summary scores. For parent HRQOL, we will similarly assess the intervention effects on the PedsQL^™^ Family Impact Module 36 items, eight subscales, and summary scores (overall total score, Parent HRQOL Summary score, and Family Functioning Summary score).^45^ To accommodate the longitudinal data collection for the HRQOL outcomes, we will use generalized linear mixed models that include main effects for time, study arm, and the interaction, to estimate timepoint-specific intervention effects. We will also evaluate the effect of the intervention on the remaining outcomes.

##### Interim analyses {21b}

Interim analyses will not be conducted during this trial.

##### Methods for additional analyses (e.g. subgroup analyses) {20b}

We will conduct mediation analysis to evaluate relationships between parent activation and the other secondary and exploratory outcomes.^57^ We will also evaluate relationships between FCR attendance and the other outcomes. For example, the intervention effect on HRQOL may be mediated by FCR attendance with a dose-response relationship. Thus, if we find a positive intervention effect on HRQOL, we will explore FCR attendance mediation using similar methods. We anticipate that the intervention effect on the following outcomes will be mediated by FCR attendance with a dose-response relationship: parent experience, family-centered care, parent activation, parent HRQOL, length of stay, and breastmilk feeding.

We will explore heterogeneity of the treatment effects, using rigorous analyses based on including interaction terms for the candidate effect modifier and the intervention effect term(s). Candidate effect modifiers will include the ones presented in Table 3 that we have either prior evidence from our pilot study and/or a strong theoretical rationale to anticipate that they may be associated with heterogeneity. For these terms, each will be evaluated without correction for multiple discovery. Other candidate effect modifiers will be evaluated as part of a comprehensive examination of intervention effects and for these, we will control the false discovery rate at 10%.

We will decompose the FCR parent attendance outcome measure by the type of attendance to separate the in-person and virtual components. We will explore the type of attendance as an outcome variable and as a predictor of the secondary outcomes. We will include a statistical exploration within the intervention arm for whether higher virtual FCR attendance is associated with differences in in-person FCR attendance. Additionally, we will fit regression models for each secondary outcome that simultaneously include measures of both virtual and in-person FCR attendance, allowing us to assess and compare whether the incremental benefits of higher FCR attendance are similar between both types of attendance.

##### Methods in analysis to handle protocol non-adherence and any statistical methods to handle missing data {20c}

The primary analysis strategy – a modified intention-to-treat analysis – differs from intention-to-treat only in that it will not require replacing missing data with imputed values. This ‘complete-case’ analysis strategy assumes that missingness is at random. Sensitivity analysis using multiple imputation will be performed to assess the potential impacts of nonignorable missingness and alternative approaches for handling infants whose disposition is not to the home and thus for whom the outcome may not be as applicable. In particular, for the few infants who transfer to another unit or hospital, the parent-reported outcomes and the 30-day revisit/readmission outcomes are of limited applicability. Our modified intention-to-treat analysis will include these subjects, but alternative analysis that excludes such outcomes from infants not discharged to home would be warranted. We will also estimate alternative treatment effects, such as per-protocol and as-treated.

##### Plans to give access to the full protocol, participant level-data and statistical code {31c}

**Access to a deidentified dataset and code may be available upon request to the principal Investigator** after completion of the study and publication of accompanying manuscripts.

#### IMPLEMENTATION EVALUATION

We will apply a pragmatic use of the five dimensions of the RE-AIM framework^35,36^ to conduct an intervention implementation evaluation within this trial. We will use a mixed methods approach with a convergent design.^58^ Table 4 shows how we will use quantitative and qualitative items relevant to each RE-AIM dimension.

##### Quantitative phase:

We will use descriptive statistics to summarize infant-, parent-, and family-level characteristics. We will analyze all available data and conduct sensitivity analyses using multiple imputation approaches for missing data.

##### Qualitative phase:

Qualitative data collection will include parent surveys and interviews. The previously described parent surveys will include a free-text response question inviting parents to provide additional thoughts or feedback about their NICU experience. We will also conduct in-depth interviews with a sample of adult parents and NICU providers (e.g., nurses, physicians, social workers). Parents will include those from the trial intervention arm. We will use convenience sampling followed by purposive sampling^59^ to ensure diversity of intervention use, role, and demographics. We will interview ~30 individuals, concluding when we reach thematic saturation. Parent recruitment will occur within two weeks after NICU discharge. Provider recruitment will occur during the last three months of the trial. One-on-one interviews will last ~45 minutes. Interviews will be audio recorded, professionally transcribed and deidentified, and reviewed for accuracy. Interviewers will maintain notes with contextual observations and cues. Participants will receive a $50 gift card.

We will use thematic analysis. Four research team members will independently memo and code the initial ten survey free-text responses and three interview transcripts using a priori codes pertaining to the RE-AIM^35^ dimensions while identifying emergent codes. We will then meet to discuss the coding structure and new topics from inductive coding. We will subsequently independently memo and code 2-5 transcripts and texts and meet again to discuss code application, refine and add codes, develop categories, and revise the interview guide. This process will be repeated with every 2-5 transcripts and texts. We will revisit prior transcripts as new codes are identified and identify linkages and patterns between the codes, which will become analytic themes. This iterative process will continue until the data coalesce around similar themes. Trustworthiness will be enhanced using interviewee and stakeholder team respondent validation on the themes and a team journal audit trail to document the qualitative procedures. We will use ATLAS.ti^60^ to organize the data.

##### Integration:

We will use a convergent design.^58^ We will compare quantitative and qualitative data using a matrix to identify congruent and divergent results. Identified discrepancies between the quantitative and qualitative findings will be resolved via a reexamination of the existing databases to gain additional insight.^58^ Should discrepancies remain that require further inquiry, we will conduct additional interviews to explore these discrepancies. We will report the merged data using narrative integration and joint display.

#### OVERSIGHT AND MONITORING

##### Composition of the coordinating centre and trial steering committee {5d}

The primary research team members will meet weekly to facilitate the day-to-day conduct of the trial. The principal investigator will have primary responsibility of all aspects of conducting the trial. The research team members will partner with a stakeholder engagement team throughout the trial.

###### Stakeholder engagement team:

A stakeholder team of parents, nurses, nurse practitioners, and neonatologists were engaged to design the trial (e.g., select trial outcomes). The stakeholder team will remain engaged throughout the trial process to conduct a relevant, acceptable, and effective intervention study.^61^ Engagement sessions will be in person when possible and otherwise via videoconference. Pre-trial engagement procedures will include refinement of the intervention procedures (e.g., workflows), refinement of documents (e.g., training materials), and assistance with NICU provider trainings. During the trial, the stakeholder team will convene monthly to review intervention subscription, intervention adherence (number of telehealth invitations sent among number of subscribed families), and survey response rates; address potential challenges; and discuss perceptions and experiences. At the conclusion of the trial, the stakeholders will assist with member checking the qualitative analysis, interpreting the data, disseminating the trial findings via publications and presentations, and developing future directions. Stakeholders receive $30/hour^62^ in gift cards for the team sessions.

##### Composition of the data monitoring committee, its role and reporting structure {21a}

This study involves no more than minimal risk. A Data Safety Monitoring Plan will be used for this study as a protection measure. We will convene an Independent Monitoring Committee consisting of three pediatric healthcare providers not associated with the study. The committee will review cumulative study data to evaluate safety, study conduct, validity, and data integrity.

##### Adverse event reporting and harms {22}

Adverse events will be collected by the principal investigator and forwarded immediately to the Independent Monitoring Committee. Additional individuals who will monitor patient safety will include the research assistants and NICU care team members. Events determined by the Independent Monitoring Committee to be unanticipated, serious, and possibly related to the study intervention will be reported to the appropriate monitoring agencies, including the University of California Davis Institutional Review Board and the NIH, within 10 days. Adverse events that are determined to be unrelated problems will be reported per Institutional Review Board policy at the time of continuing review.

##### Frequency and plans for auditing trial conduct {23}

The committee will meet to independently review outcomes on a quarterly basis and as needed based on any reported complications. The safety monitoring will begin when the trial enrollment begins. The committee will complete quarterly reports detailing the study progress, any adverse events, and any protocol deviations.

##### Plans for communicating important protocol amendments to relevant parties (e.g. trial participants, ethical committees) {25}

**Any protocol amendments will be discussed with and approved by the study sponsors and the** University of California Davis Institutional Review Board, as relevant.

##### Dissemination plans {31a}

**Trial results will be disseminated at national and international conferences and through peer-reviewed research publications.** All peer-reviewed manuscripts that result from this trial will be submitted to the PubMed Central digital archive.

## Discussion

This trial is the first randomized controlled trial to date to evaluate the impact of using virtual FCR in the NICU on parental and infant outcomes. This trial builds on prior research supporting the feasibility and acceptability of using telehealth for virtual FCR,^21–24^ including our team’s pilot data on virtual FCR in the NICU.^24^ We now test our intervention with adequate power for hypothesis testing of the primary and secondary outcomes, and we will assess intervention effects as well as heterogeneity of the treatment effects. Our proposed hybrid type 1 effectiveness-implementation approach^63^ that additionally examines implementation outcomes will speed the translation of our research findings to facilitate the dissemination of this telehealth solution.

Knowledge gained from the mixed methods RE-AIM implementation evaluation will help us understand the contextual factors that influence implementation of our intervention so that diverse populations can equitably benefit from our telehealth advancement. Underserved populations face barriers to equitably accessing telehealth service,^64–66^ and use of the RE-AIM framework as proposed in our trial is a strategy to address telehealth intervention reach and adoption issues.^36^

Virtual FCR is a strategy that acknowledges the realities of parents’ lives and the barriers they face to engage in care. Our intervention addresses healthcare inequities and allows FCR to actually be family-centered. Ultimately, this research has potential to ensure NICU parents can engage in FCR to increase the delivery of best practice and to enhance clinical outcomes and family quality of life.

## Figures and Tables

**Table 1:** Trial outcome measures

Outcome Name	Outcome Type	Data Source
Parent FCR attendance	Primary	Obtained from observation
Parent experience	Secondary	Child HCAHPS^40,41^ parent survey (2 items)
Family-centered care	Secondary	FACCE^42^ parent survey (10 items)
Parent activation	Secondary	P-PAM^43,44^ parent survey (10 items)
Parent HRQOL	Secondary	PedsQL^™^ Family Impact Module^45^ (36 items)
Length of stay, days	Secondary	EHR
Breastmilk feeding	Secondary	EHR and parent report
Growth failure	Secondary	EHR
Growth velocity	Exploratory	EHR
Adverse events/errors	Exploratory	EHR and solicited reports
30-day revisit	Exploratory	EHR and parent report
30-day readmission	Exploratory	EHR and parent report
Temperature instability	Exploratory	EHR
Central line-associated bloodstream infection	Exploratory	EHR
Central line days	Exploratory	EHR
Antibiotic days	Exploratory	EHR

FCR – family-centered rounds

HRQOL – health-related quality of life

EHR – electronic health record

**Table 2:** Trial schedule of enrollment, intervention, and assessments

	Study Period						
	Enrollment	Randomization	Intervention period (NICU hospitalization)	Follow-up (days post discharge)			
	−*t*	0		0	30	60	90
**Eligibility screen**	X						
**Randomization**		X					
**Assessments**							
***Covariates***							
Neonatal characteristics	X		X				
Parental characteristics	X		X	X			
***Primary Outcome***							
Parent FCR attendance			X				
***Secondary Outcomes***							
Parent experience (Child HCAHPS)				X			
Family-centered care (FACCE)				X			
Parent activation (P-PAM)				X			
Parent health-related quality of life (PedsQL^™^ Family Impact Module)				X	X	X	X
NICU length of stay, days				X			
Breastmilk feeding			X	X			X
Growth failure				X			
***Exploratory Outcomes***							
Growth velocity				X			
Adverse events/errors			X				
30-day revisit					X		
30-day readmission					X		
Temperature instability			X				
Central line-associated bloodstream infection			X				
Central line days			X				
Antibiotic days			X				

FCR – family-centered rounds

**Table 3:** Anticipated interactions between subject characteristics and the intervention effect

Characteristic	Anticipated Outcomes Impacted and Direction ^a,b,c,d,e,f,g,h^	Rationale
Residence-to-NICU distance	Families with greater residence-to-NICU distance will have greater intervention effect compared to families with shorter distance ^a,e^	The intervention will be particularly helpful for parents with more barriers to travel
California HPI	Families with lower HPI (from neighborhoods with fewer resources) will have greater intervention effect compared to those with higher HPI ^a,e^	The intervention will be particularly helpful for families from neighborhoods with fewer resources
Parent race and ethnicity	Parents of minority races and ethnicities will have greater intervention effect compared to non-Latinx White parents ^a,g^	Using our pilot trial^24^ data, among the control arm, the weighted incidence rate for the FCR parent attendance proportion for racial and ethnic minority subjects (relative to non-Latinx White subjects) was 0.32 (95% CI 0.20–0.50). Among the intervention arm, the weighted incidence rate became 0.68 (95% CI 0.56–0.83). The rigorous comparison of these two ratios revealed that the IRR for minority subjects*intervention interaction was 2.15 (95% CI 1.30–3.56) ^i^
Parent transportation security	Families with transportation insecurity will have greater intervention effect compared to families with access to adequate transportation ^a,e^	The intervention will be particularly helpful for parents with more barriers to travel
Other children dependents	Parents with other children dependents will have greater intervention effect compared to parents without other children dependents ^a,e^	The intervention will be particularly helpful for parents with other children dependents; virtual FCR will mitigate barriers to attend FCR due to competing childcare responsibilities
Parent computer/smart device access	Parents lacking computer/smart device access will have lower intervention effect compared to those with access ^a^	Parents lacking computer/smart device access will have fewer resources to attend FCR – either in-person or virtually
Parent internet access	Parents with no/limited internet access will have lower intervention effect compared to those with high speed access ^a^	Parents with no/limited internet access will have fewer resources to attend FCR – either in-person or virtually
Parent digital literacy score	Parents with lower digital literacy will have lower intervention effect compared to those with high digital literacy ^a^	Parents with lower digital literacy will have greater challenges in using the intervention

NICU – neonatal intensive care unit

HPI – healthy places index

FCR – family-centered rounds

IRR – incidence rate ratio

Primary and secondary outcomes:

a family-centered rounds attendance,

b parent experience,

c family-centered care,

d parent activation,

e parent health-related quality of life,

f length of stay,

g breastmilk feeding,

h neonatal growth

i unpublished data

**Table 4:** Quantitative and qualitative items for each RE-AIM dimension

Dimension	Quantitative Items	Qualitative Items
**Reach**		
	• % excluded and characteristics • Characteristics of parent(s) who subscribe to use virtual FCR among intervention arm subjects	• Explore factors influencing reach • Explore factors influencing intervention subscription

**Effectiveness**		
	• Between arm comparisons for trial outcomes • Heterogeneity of intervention effects	• Explore mechanisms of action for outcomes • Explore mechanisms of potential heterogeneity effects • Explore unmeasured intervention effects

**Adoption**		
	• % FCR encounters with virtual vs. in-person vs. both vs. no parent attendance • % and characteristics of virtual FCR users vs. non-users among intervention arm subjects	• Explore factors influencing parent participation • Explore factors influencing provider participation

**Implementation**		
	• % of virtual FCR attempts with technical issues • % FCR encounters with a telehealth visit initiated among encounters with subscribed parent(s) • % of weekdays with a disruption in FCR (e.g., delivery or admission)	• Explore factors influencing implementation • Implementation adaptations made • Adaptations made to NICU practices and/or policies and procedures

**Maintenance**		
	• Number per month of virtual FCR used post-trial	• Explore virtual FCR aspects sustained/modified post-trial

FCR – family-centered rounds

## Data Availability

The datasets used and/or analysed during the current study are available from the corresponding author on reasonable request.

## Abreviations

RE-AIM: Reach, Effectiveness, Adoption, Implementation, Maintenance
FCR: family-centered rounds
NICU: neonatal intensive care unit
EHR: electronic health record
SD: standard deviation
